# Bringing it all together: Wearable data fusion

**DOI:** 10.1038/s41746-023-00897-6

**Published:** 2023-08-17

**Authors:** Yunus Celik, Alan Godfrey

**Affiliations:** Department of Computer and Information Sciences, Northumbria University, Newcastle upon Tyne, UK

**Keywords:** Biomedical engineering

## Abstract

Contemporary wearables like smartwatches are often equipped with advanced sensors and have associated algorithms to aid researchers monitor physiological outcomes like physical activity levels, sleep patterns or heart rate in free-living environments. But here’s the catch: all that valuable data is often collected separately because the sensors don’t always play nice with each other, and it’s a real challenge to put all the data together. To get the full picture, we may often need to combine different data streams. It’s like putting together a puzzle of our health, instead of just looking at individual pieces. This way, we can gather more useful info and better understand health (it’s called digital twinning). Yet, to do so requires robust sensor/data fusion methods at the signal, feature, and decision levels. Selecting the appropriate techniques based on the desired outcome is crucial for successful implementation. An effective data fusion framework along with the right sensor selection could contribute to a more holistic approach to health monitoring that extends beyond clinical settings.

## Introduction

Commercial-based wearables have become an integral part of our lives, revolutionising the way we monitor and manage our health^1,2^. Devices including smartwatches, fitness trackers, respiratory bands, electrodermal response sensors, and smart insoles have the potential to enable more insightful physical examinations through high-resolution data. It’s akin to a mini clinic worn directly on our body. Many physiological outcomes can be collected from those now described as routine (e.g., heart rate, blood oxygen levels, sleep patterns) to those previously unattainable beyond a clinic or complex monitoring equipment (e.g., electrocardiogram, electrodermal activity)^3^. But here’s the thing: having all these data doesn’t necessarily give the full picture of health^4–6^. For instance, quantifying daily heart rate is useful, but without considering other outcomes such as physical activity levels or sleep quality, it may be meaningless to gauge the true impact of daily activities on overall health or treatment interventions. There is a need to move beyond isolated data points and embrace a holistic approach to monitoring. That’s where data fusion comes in. It’s the process of combining data from different sources to create a more complete picture of what’s going on.

Let’s examine two studies within *npj Digital Medicine*. The first study compared the Cardiac and Activity Monitor (CAM, https://verily.com) worn on 9 different body locations to conventional clinical measures^7^. Those devices were used to extract various features related to gait (e.g., stance time), turns (e.g., turn duration), balance (sway), and cardiovascular outcomes (e.g., heart rate variability). Findings demonstrated a strong correlation between the digital biomarkers obtained from the wearables and the conventional clinical outcomes. The second is a very recent article that combined multiple sensor readings from a Fitbit (www.fitbit.com) device worn by 22 individuals diagnosed with Pulmonary Arterial Hypertension (PAH)^8^. The study collected data to derive a range of physical activity parameters, including weekly heart rate, step rate distributions, ambulation frequency as well as fitness and health state measures. The study concluded that additional free-living physical and cardiovascular functions can provide much more detailed information than daily step counts alone via a *routine* wearable. Yet, there was a dearth of information pertaining to fusion framework (e.g., in what level data is fused / what framework is employed). This information is essential for establishing the most appropriate algorithm for the defined problem and critical to successfully implementing data fusion^9^. In this editorial, we’ll look at different scenarios and talk about the challenges and factors that need to be considered when trying to bring together data from different sources, especially wearables. The goal is to make health monitoring more complete and informative, and not just limited to clinics.

## Fusion for comprehensive health monitoring beyond clinics

The use of numerous systems and data fusion could ensure increased dimensionality of patient measurement through multiple sensor types^9^. Typically, data or sensor fusion can take place at signal level, feature level and decision level^10^. Briefly, signal-level fusion involves the utilisation of raw sensor data, while feature-level fusion incorporates extracted features from raw signals. So instead of using all the raw data, we pick out certain characteristics that are useful for understanding the patient’s health. This helps us make sense of the data in a more focused way. Alternatively, decision-level fusion often depends on the understanding of the perceived situation, which is derived from multiple sources^11^. Given the fact that each health sensor unit captures distinct physiological parameters (e.g., sweat or glucose level), feature level fusion or decision-level fusion may be more feasible to achieve holistic healthcare applications. Signal-level fusion may have limitations as the collected raw data from two different biosensors (e.g., sweat and glucose sensor) are not directly comparable. But sometimes, one can still do signal-level fusion if it makes sense. For example, if we want to estimate joint movements accurately, we can combine the raw data from sensors that measure acceleration and angular velocity. This helps us get more precise and reliable results^12^.

The following examples fall within the context of feature or decision-level fusion and could be useful to understand the concept of data fusion in wearable health monitoring. Consider the scenario that accelerometer and electrocardiogram (ECG) signals are fused to identify of abnormal heart rhythms, such as arrhythmias. An optimal approach in this case would involve employing feature-level fusion that concatenate the feature vectors from both modalities into a single feature vector. Initially, features extracted from the accelerometer signal could be used to identify the type of activity being performed (e.g., walking or sitting). Subsequently, features extracted from the ECG signal could be used to analyse the heart rate and identify any abnormalities or irregularities during those activities. This two-step process of feature extraction and interpretation from both sensor signals enables a more comprehensive assessment of the individual’s heart health. Alternatively, consider researchers conducting a study to reveal how sleep quality affects heart rate variability and stress levels during the day. Here, sleep quality from the night can be scored using actigraphy, polysomnography (PSG), and respiratory sensors (e.g., nasal airflow sensors). Then this information could be combined with galvanic skin response (GSR), body temperature, and heart rate sensor readings at the decision level (e.g., voting, averaging techniques, or Bayesian inference) to better inform patient assessment.

As technology continues to advance, wearable sensors will get smaller and become more efficient and functional i.e., capturing various health metrics that are not possible to capture today. Consequently, fusion will play an increasingly vital role in combining the outcomes of wearable biosensors. This could initiate transforming healthcare by providing comprehensive and personalized solutions for improving individual well-being and enabling proactive healthcare management. Some examples are already evident in the literature. For instance, a previous study showed that the combination of multiple measurement resources such as acceleration, force and 3D skeleton data has shown great potential in accurately identifying distinct gait patterns linked to Parkinson’s disease severity^13^. Specifically, the referenced study proposed a novel framework that relies on various feature extraction algorithms along with a multi-switch discriminator to associate observations with individual state estimations. In another study, a multimodal approach (multi-layer sensor fusion) has been employed to gain deeper insights into the disparities between the affected (paretic) and unaffected (non-paretic) sides of stroke survivors^14^. That approach encompasses the analysis of spatiotemporal factors, joint kinematics, and muscle activation, aiding in a comprehensive understanding of the differences. Although these frameworks have proven to be useful and yield the desired outputs, there are specific challenges that must be addressed. The primary limitation lies in the varying sampling frequencies of biosensors, which makes synchronization more challenging. This issue could be particularly problematic in health monitoring scenarios, especially in experiments that require simultaneous measurements, such as monitoring coordination between different body parts. Addressing these challenges is critical, and it is beneficial to consider various factors that contribute to the effective use of data/sensor fusion in health monitoring. By taking these factors into account, researchers could enhance the potential to overcome the challenges associated with fusion techniques in health monitoring applications, paving the way for use in low-resource settings or even remote deployment in the home.

## Data fusion considerations

When implementing data fusion in healthcare applications, several factors must be considered to ensure optimal outcomes and reliable results. Sensor selection is the primary factor as each sensor should be chosen based on its relevance to the target outcome and accuracy^15^. The latter is especially important in healthcare applications, and it requires calibration on a regular basis. This is mainly because, over time, sensors can drift from their initial calibration due to various factors such as environmental conditions, wear and tear, or electronic variations. Calibration helps correct any deviations and ensures the sensor’s readings align with known standards or reference measurements. It also helps reduce measurement errors, inconsistencies, and variability, thereby improving the reliability of the sensor’s output^16^. IoT connectivity, such as Bluetooth or Wi-Fi, in wearable biosensors, can offer several advantages in terms of data fusion. As an example, wearable sensors equipped with Internet of Things (IoT) connectivity have the capability to transmit live data directly to compatible platforms. This allows for real-time data fusion, enabling remote monitoring through seamless integration. However, robust security measures must be implemented to safeguard patient data and ensure compliance with applicable privacy regulations^17^.

Another consideration is selection of fusion methodology. Each fusion approach has its strengths and limitations, and selecting the most appropriate methodology requires careful consideration of the desired outcomes. For example, feature-level fusion requires the selection of features that relies on domain knowledge. If the chosen features do not adequately represent the underlying physiological processes or fail to capture the full complexity of the data, the fusion results may be limited or less accurate. It’s like trying to solve a puzzle with missing pieces or even by using the wrong pieces. Or different experts may prioritise different features, leading to potential discrepancies in the fusion outcomes. In general, it is essential to establish a verification, analytical, and clinical validation (V3) process to ensure that the fusion output is suitable for its intended purpose^18^.

## Conclusion

Fusion techniques stemming from free-living wearable health monitoring offer a promising avenue for achieving a more comprehensive understanding of an individual’s health. The examples presented highlight the potential of data fusion in capturing complex physiological processes and extracting meaningful insights from multiple health metrics. Whether through feature-level fusion or decision-level fusion, combining information from distinct sensor systems enhances the dimensionality of measurements and increase our understanding in various areas. As wearable technology continues to advance, fusion techniques will play a pivotal role in transforming healthcare by providing personalized and comprehensive solutions for improving individual well-being and enabling proactive healthcare management.
